# Characterizing electronic noise interference from the external laser positioning system on a radiation therapy MRI simulation scanner

**DOI:** 10.1002/acm2.70731

**Published:** 2026-08-06

**Authors:** Lucas McCullum, Yao Ding, Clifton D. Fuller, Brian A. Taylor

**Affiliations:** ^1^ UT MD Anderson Cancer Center UTHealth Houston Graduate School of Biomedical Sciences Houston Texas USA; ^2^ Department of Radiation Oncology The University of Texas MD Anderson Cancer Center Houston Texas USA; ^3^ Department of Radiation Physics The University of Texas MD Anderson Cancer Center Houston Texas USA; ^4^ Department of Imaging Physics The University of Texas MD Anderson Cancer Center Houston Texas USA

**Keywords:** external laser positioning system, magnetic resonance imaging, MRI simulation, quantitative imaging

## Abstract

**Background:**

Magnetic resonance imaging (MRI) for radiation therapy treatment planning is currently being used in many anatomical sites to better visualize soft tissue landmarks, a technique known as an MRI simulation. A core component of modern MRI simulation configurations are the use of external laser positioning systems (ELPS) to help set up the patient. Though necessary for accurate and reproducible patient setup, the ELPS, if left on during imaging, may interfere negatively with image quality due to leaking electronic noise, of which MRI is sensitive to.

**Purpose:**

It is currently unknown whether this leakage of electronic noise may further affect quantitative values derived from clinically employed relaxometric, diffusion, and fat fraction sequences. Therefore, in this study, we aim to characterize the impact of MRI simulation lasers on general image quality and quantitative imaging accuracy.

**Methods:**

First, a cine acquisition was used to visualize the real‐time changes in image signal‐to‐noise ratio (SNR) from when the ELPS was deactivated to activated. To validate this effect quantitatively, the SNR was measured using the American College of Radiology (ACR) recommended T1‐weighted protocol in a homogeneous phantom with the integrated body, 18‐channel UltraFlex small, 18‐channel UltraFlex large, 32‐channel spine, and 16‐channel shoulder coils. Next, a geometric distortion algorithm was tested in two vendor‐provided phantoms while using the integrated body coil and the ACR Large Phantom protocol was tested. Finally, a series of quantitative MRI scans were performed using a CaliberMRI Model 137 Mini Hybrid phantom to validate quantitative T1, T2, and ADC while a Calimetrix PDFF‐R2* phantom was used for quantitative PDFF and R2*. All scans were performed with both the ELPS both deactivated and activated.

**Results:**

Visible electronic noise artifacts were seen when using the integrated body coil when the ELPS was activated on the cine acquisition which led to a two‐fold decrease in SNR using the ACR protocol, however geometric distortion quantification was not affected. This SNR drop was not seen when using the remaining tested coils. Degradation in image intensity uniformity, percent signal ghosting, and low contrast object detectability was seen during ACR Large Phantom testing using the 20‐channel Head/Neck coil. Concordance across quantitative MRI values was similar when the ELPS was both deactivated and activated while a consistent increase in standard deviation inside the ADC vials was seen when the ELPS was activated.

**Conclusions:**

The extra noise induced from the activation of the ELPS during imaging should be avoided due to its potential to unnecessarily increase image noise. This is particularly true when conducting mandatory quality assurance testing for image quality and geometric distortion which utilize the integrated body coil which is most susceptible to ELPS‐induced noise. Clear clinical guidelines should be implemented to make this issue known to the MRI technologists, physicists, and other relevant staff using an MRI with a supplementary ELPS for patient alignment.

## 1 INTRODUCTION

Magnetic resonance imaging (MRI) for radiation therapy treatment planning is currently being used in many anatomical sites to better visualize soft tissue landmarks, a technique known as MRI simulation. A core component of modern MRI simulation configurations are the use of external laser positioning systems (ELPS) to help set up the patient. Though necessary for accurate and reproducible patient setup, these devices, if left on during imaging, may interfere negatively with image quality due to leaking electronic / radiofrequency (RF) noise^1^, of which MRI is sensitive to^2^. These RF interference artifacts, also referred to as “zipper” artifacts due to their often zipper‐like appearance, align along the phase encoding direction and orthogonal to the frequency encoding direction^2^. They should be differentiated from k‐space spikes, or “corduroy” / “herringbone” artifact, where stripes are introduced uniformly throughout the whole image with angle and periodicity directly related to the location of the spike in k‐space^3^. The frequency of the interference can be approximated by its location in the image along with the receiver bandwidth. Single frequency components will appear as thin lines, while more broadband frequency sources will appear as wider lines or even global image noise if the range of frequencies is large enough. The blips along the phase encoding direction that create the zipper‐like appearance are due to the periodicity of the RF interference and can be approximated using the pixel offset of the blips and the repetition time (TR) of the acquisition.

The most common ELPS used in MRI simulation is manufactured by LAP (LAP of America Laser Applications L.L.C.; Boynton Beach, FL, USA). These devices are noted as MR Conditional in the user manual with the requirement that both the laser positioning system and monitor are switched off during MR scanning. This can be done from a master switch which should be included in the clinical workflow of MRI technologists. If this is not done, then reciprocal effects may occur between the LAP system and the MR scanner due to its 50–60 Hz operating frequency as noted in the user manual. Contrary to initial thoughts, it is not sufficient to simply turn off the lasers. Instead, the entire system must be deactivated which would deactivate all lights seen on the system including that of the monitor. Therefore, the intended clinical workflow for the system would be to activate it during patient positioning, ensure its deactivation during scanning, and re‐activate it for the next patient. However, cumbersome placement of the master switch or improper training of the MRI technologists can lead to scans being conducted while the ELPS is still powered on.

At our institution, we have had instances where quality assurance and periodic maintenance noise tests have failed due to the ELPS being activated, while passing when the ELPS was deactivated again. This unintended discovery led us to notice image quality concerns with deactivation of the ELPS system at our institution during MRI simulation scans due to lack of education and logistical burdens regarding the placement of the deactivation switch. This issue is of primary concern since preventable noise may be incorporated into the images on our MRI simulation scanners. Further, our institution frequently incorporates quantitative imaging including T1 and T2 mapping using multi‐dynamic multi‐echo (MDME) sequences^4^, apparent diffusion coefficient (ADC) maps^5^ from diffusion‐weighted imaging (DWI), and quantitative Dixon producing R2* and proton density fat fraction (PDFF) maps^6^, ^7^ into our scanning workflow and it is currently unclear whether the activation of the ELPS during scanning affects the quantitative values of these scans in terms of bias or repeatability. Another unknown is whether the artifacts are of considerable concern only when using the integrated body coil as in the quality assurance and period maintenance tests, or when clinical multi‐channel array coils are used as well. Therefore, the purpose of this paper was to determine: 1) the direct effect of the activated ELPS on image quality through image artifacts and presence of noise, 2) the artifact's presence across different coil types used, and 3) the subsequent effect of these conditions on quantitative MRI bias and repeatability.

## 2 METHODS

### 2.1 Phantom assessment

A vendor‐provided homogeneous phantom in a container with 1900 mL of “Solution N” (3.75 g NiSO_4_ x 6H_2_O and 5 g NaCl per 1,000 g H_2_O) was used for signal‐to‐noise ratio (SNR) assessment and artifact identification. The American College of Radiology (ACR) Large Phantom was used to perform the wide range of tests defined in their MRI Accreditation Program. The CaliberMRI Model 137 Mini Hybrid Phantom (CaliberMRI; Boulder, CO, USA) was used as a reference for National Institute of Science and Technology (NIST)‐traceable T1, T2, and ADC values. Additionally, the Calimetrix Model 725 PDFF‐R2* phantom^8^ (Calimetrix; Madison, WI, USA) was used to validate quantitative R2* and PDFF values. The bore temperature was approximately 20°C across all acquisitions following assessment with an analog thermometer places near the bore which remained stable across acquisitions with included break time of several minutes between repetitions to prevent substantial heating. In line with our current clinical workflow for head and neck cancer patients, two 18‐channel UltraFlex small and spine coil elements 1–3 were used.

Additionally, the effects of different coils (integrated body coil, 20‐channel BioMatrix Head/Neck Tiltable CoilShim—TCS, 18‐channel UltraFlex small, 18‐channel UltraFlex large, 32‐channel spine coil elements 1–4, and 16‐channel shoulder) on the presence of the RF interference artifact was studied. Since our institution measures geometric distortion using the integrated body coil, we decided to test that workflow with the ELPS both deactivated and activated using the QUASAR™ GRID^3D^ Image Distortion Phantom (Modus Medical Devices; London, Ontario, Canada) which is a smaller phantom of 152 × 182 × 190 mm^3^ consisting of 1765 control points and accompanying vendor‐provided automated geometric distortion analysis.

### 2.2 MRI acquisition parameters

All scans were performed on a 3T Siemens Vida (XA60; Siemens Healthcare; Erlangen, Germany) MRI‐simulation scanner equipped with an ELPS (DORADOnova MR3T; LAP of America Laser Applications L.L.C.; Boynton Beach, FL, USA). Cine images to assess real‐time changes in image quality during ELPS activation were acquired using fast imaging with 2D steady‐state precession (TrueFISP^9^, ^10^) sequence with a field‐of‐view = 256 × 256 × 15 mm^3^, matrix size = 128 × 128 × 1, reconstructed voxel size = 1 × 1 × 15 mm^3^, flip angle = 29 degrees, TR = 160 ms, TE = 1 ms, GeneRalized Autocalibrating Partially Parallel Acquisitions (GRAPPA^11^) factor = 2, receive bandwidth = 1502 Hz/Px, number of dynamics = 513, and scan time = 1 min 6 s. A 3D T1‐weighted FLASH sequence was applied to image the QUASAR geometric distortion phantom with FOV = 256 × 256 × 160 mm^3^, matrix = 256 × 256 × 160, reconstructed voxel size = 1 × 1 × 1 mm^3^, TR = 6.2 ms, TE = 2.7 ms, flip angle = 20 degrees, receive bandwidth = 399 Hz/Px, number of averages = 2, and scan time = 4 min 25 s. The standard ACR T1‐weigthed and T2‐weighted sequences as described in the MRI Accreditation Program manual were used to perform the tests on the ACR Large Phantom using the 20‐channel Head/Neck coil. The T1‐weighted sequence was also used to determine the SNR of each coil type both while the ELPS was activated and deactivated. Both the ACR T1‐weighted and T2‐weighted sequences were a 2D spin echo with FOV = 250 × 250 × 105 mm^3^, matrix = 256 × 256 × 11, reconstructed voxel size = 0.98 × 0.9 × 5 mm^3^, distance factor = 100%, and receive bandwidth = 250 Hz/Px. The T1‐weighted sequence had TR = 500 ms, TE = 20 ms, and scan time = 2 min 10 s while the T2‐weighted sequence had TR = 2000 ms, TE = 80 ms, and scan time = 8 min 30 s.

For this study, three clinically deployed quantitative imaging techniques were tested to determine the impact of scanning during ELPS activation on their accuracy and repeatability. First, for quantitative T1 and T2 relaxometry mapping, a 2D multi‐dynamic multi‐echo (MDME) sequence^12^ was acquired. Second, for quantitative ADC values, a 3D motion correction with radial blades (BLADE) DWI sequence^13^ was used. Finally, a 3D quantitative Dixon (q‐Dixon) sequence was used to determine quantitative R2* and PDFF. The respective 2D or 3D Siemens built‐in distortion correction was added to each sequence to improve geometric fidelity and reduce repeatability bias. These sequences were acquired using two 18‐channel UltraFlex small coils anterior to the phantom and spine coil elements 1–4 posterior to the phantom with detailed acquisition parameters shown in Table 1. The repeatability of the quantitative values was determined by conducting five repetitions of each quantitative MRI sequence using their respective phantom (MDME and BLADE DWI for the CaliberMRI phantom and q‐Dixon for the Calimetrix phantom) while the ELPS was both activated and deactivated.

**TABLE 1:** The acquisition parameters for the quantitative MRI sequences used in this study. Note that some parameters may not be valid for the specified sequence which is indicated by a dashed line. Abbreviations: EPI = echo planar imaging, ETL = echo train length, GRAPPA = GeneRalized Autocalibrating Partially Parallel Acquisitions, TE = echo time, TR = repetition time.

	MDME	BLADE DWI	q‐Dixon
Field‐of‐View (mm^3^)	256 × 256 × 200	256 × 256 × 208	256 × 256 × 248
Matrix Size	256 × 256 × 50	128 × 128 × 52	128 × 128 × 124
Reconstructed Voxel Size (mm^3^)	1 × 1 × 3	2 × 2 × 4	2 × 2 × 2
Flip Angle (°)	150	110	4
TR (ms)	7310	5900	7.95
TE (ms)	19 / 94	38	1.03 / 2.15 / 3.27 / 4.39 / 5.51 / 6.63
Echo Spacing (ms)	9.38	8.38	–
GRAPPA Factor	3	3	–
Receive Bandwidth (Hz/Px)	210	651	1116
Additional Notes	ETL = 12, N_dynamics_ = 4, Slice Gap = 0.3 mm	b‐values (averages) = 0 (1) / 800 (6) s/mm^2^, ETL = 18, EPI Factor = 3	–
Scan Time (m:ss)	5:31	5:50	3:03

### 2.3 Post‐processing and statistical analysis

All relevant analysis concerning statistical methods were formulated using the guidelines for reporting Statistical Analyses and Methods in the Published Literature (SAMPL^14^) and all computations unless otherwise specified were performed using Python 3.12.10. The image SNR was determined using Method 4 from the National Electrical Manufacturers Association (NEMA) Standard Publication MS 1–2008. Specifically, this was done by determining the ratio of the mean signal region‐of‐interest (ROI) in the homogeneous region and the corner noise ROIs with a Gaussian to Rayleigh distribution correction^15^. Quantitative SNR determination was made for the different coil combination images using ImageJ^16^. The mean and standard deviation for four repeated SNR measurements at different noise locations were then determined and tested for significance using an unpaired Student's t‐test assuming equal variances with a significance level of 0.05. All ACR Large Phantom analysis was done following the ACR MRI Accreditation Program guidelines from October 19, 2022. The quantitative T1 and T2 values from the MDME were reconstructed using SyMRI StandAlone 11.3.11 (SyntheticMR AB; Linkoping, Sweden) while the ADC, PDFF, and R2* images were taken directly from the scanner reconstruction. For all the quantitative MRI sequences, a region‐of‐interest (ROI) was created for each vial of the respective phantom with a slight margin to avoid the inclusion of ringing artifacts. All ROIs were created in 3D Slicer^17^ (https://www.slicer.org) and exported in NIfTI format^18^. After extracting the quantitative imaging values from each vial of the phantom, Lin's Concordance Correlation Coefficient (LCCC^19^) was used to assess direct agreement between the mean measured vial values and the phantom reference values combined across all repetitions. Next, Bland‐Altman plots^20^ were used to assess quantitative values that exceeded an expected 95% limit of agreement threshold using the combined values across all repetitions. A linear fit was computed for reference acquisitions against nominal phantom reference values. Additionally, the standard deviation and subsequent coefficient‐of‐variation (CoV) inside each vial was computed.

## 3 RESULTS

### 3.1 Non‐quantitative imaging analysis

**FIGURE 1:**
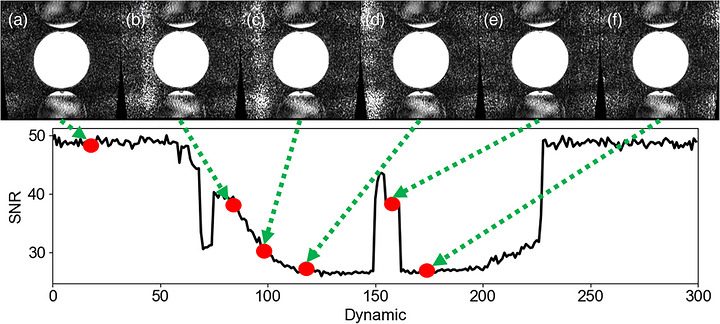
The presentation of the RF interference artifact and its respective SNR change induced by the activation of the ELPS throughout the cine acquisition.

The progression of the RF interference artifact in the cine acquisition is shown in Figure 1. Initially, no artifact was seen in Figure 1(a). However, once the ELPS was activated, an RF interference artifact was seen to the left of the phantom Figure 1(b) and gradually shifts towards the left throughout each dynamic of the cine in Figure 1(c) and (d), effectively reducing the SNR by half. After full activation and when the RF band begins to wrap around, only skinnier lines with blips are seen throughout the image in Figure 1(e) indicative of a narrower RF band. Finally, after wrapping around, a brief and subtle RF interference artifact similar to Figure 1(d) was seen on the right side of the image in Figure 1(f) before returning to Figure 1(a) once the ELPS has been deactivated.

To determine the effect of coil choice, the SNR in the homogenous phantom when the ELPS was deactivated and compared to when it was activated across different coil types is shown in Table 2. The integrated body coil was significant with an SNR drop from 14 to 8 followed by the spine elements with a drop from 79 to 69 while the rest of the coil combinations tested were not significant.

**TABLE 2:** A demonstration of the change in mean (standard deviation) SNR when the ELPS was deactivated and activated in a homogeneous phantom across different coil types.

	SNR (ELPS Deactivated)	SNR (ELPS Activated)	P‐Value
Integrated body coil	14 (0.3)	8 (0.8)	<0.0001
20‐Channel BioMatrix Head/Neck TCS	100 (14)	90 (5)	0.47
18‐Channel UltraFlex Small	219 (35)	215 (41)	0.91
18‐Channel UltraFlex Large	217 (23)	203 (18)	0.57
32‐Channel Spine Elements 1–4	79 (1.2)	69 (1.3)	0.015
16‐Channel Shoulder	154 (12)	157 (16)	0.87

The results of the ACR MRI Accreditation Program tests with both the ELPS deactivate and activated are shown in Table 3. A 5% decrease in the image intensity uniformity in the ACR T2‐weighted sequence when the ELPS was activated was seen, though a slight increase (91.19 vs. 90.67%) was seen in the ACR T1‐weighted sequence. The percent signal ghosting also increased by a factor of 4.5 when the ELPS was activated leading to a failed low contrast object detectability test for the ACR T2‐weighted sequence.

**TABLE 3:** The ACR MRI Accreditation Program test results when the ELPS was deactivated and activated using the ACR Large Phantom. Abbreviations: Ax = axial, Diag = diagonal, LR = lower right, Sag = sagittal, UL = upper left.

	ELPS Deactivated	ELPS Activated
Sag geometric accuracy (148 ± 3 mm)	**Height**: 146.5 mm	**Height**: 145.2 mm
Ax geometric accuracy (190 ± 3 mm)	**Slice 1 Height**: 189.5 mm	**Slice 1 Height**: 189.8 mm
	**Slice 1 Width**: 189.5 mm	**Slice 1 Width**: 189.5 mm
	**Slice 5 Height**: 189.5 mm	**Slice 5 Height**: 189.8 mm
	**Slice 5 Width**: 188.8 mm	**Slice 5 Width**: 189.2 mm
	**Slice 5 Diag Down**: 188.9 mm	**Slice 5 Diag Down**: 188.5 mm
	**Slice 5 Diag Up**: 189.0 mm	**Slice 5 Diag Up**: 188.3 mm
High contrast spatial resolution (≤ 1 mm)	**T1 UL**: 1 mm	**T1 UL**: 1 mm
	**T1 LR**: 1 mm	**T1 LR**: 1 mm
	**T2 UL**: 1 mm	**T2 UL**: 1 mm
	**T2 LR**: 1 mm	**T2 LR**: 1 mm
Slice thickness accuracy (5 ± 1 mm)	**T1**: 5.01	**T1**: 4.59
	**T2**: 5.07	**T2**: 4.88
Slice Position Accuracy (≤ 7 mm)	**T1 Slice 1**: ‐1.95	**T1 Slice 1**: 4.15
	**T1 Slice 11**: ‐1.06	**T1 Slice 11**: 3.91
	**T2 Slice 1**: ‐2.02	**T2 Slice 1**: 3.23
	**T2 Slice 11**: ‐0.80	**T2 Slice 11**: 3.85
Image intensity uniformity (≥ 85%)	**T1**: 90.67%	**T1**: 91.19%
	**T2**: 95.56%	**T2**: 90.88%
Percent signal ghosting (≤ 3%)	0.0038%	0.017%
Low contrast object detectability (≥ 37)	**T1**: 39	**T1**: 40
	**T2**: 37	**T2**: 35

**FIGURE 2:**
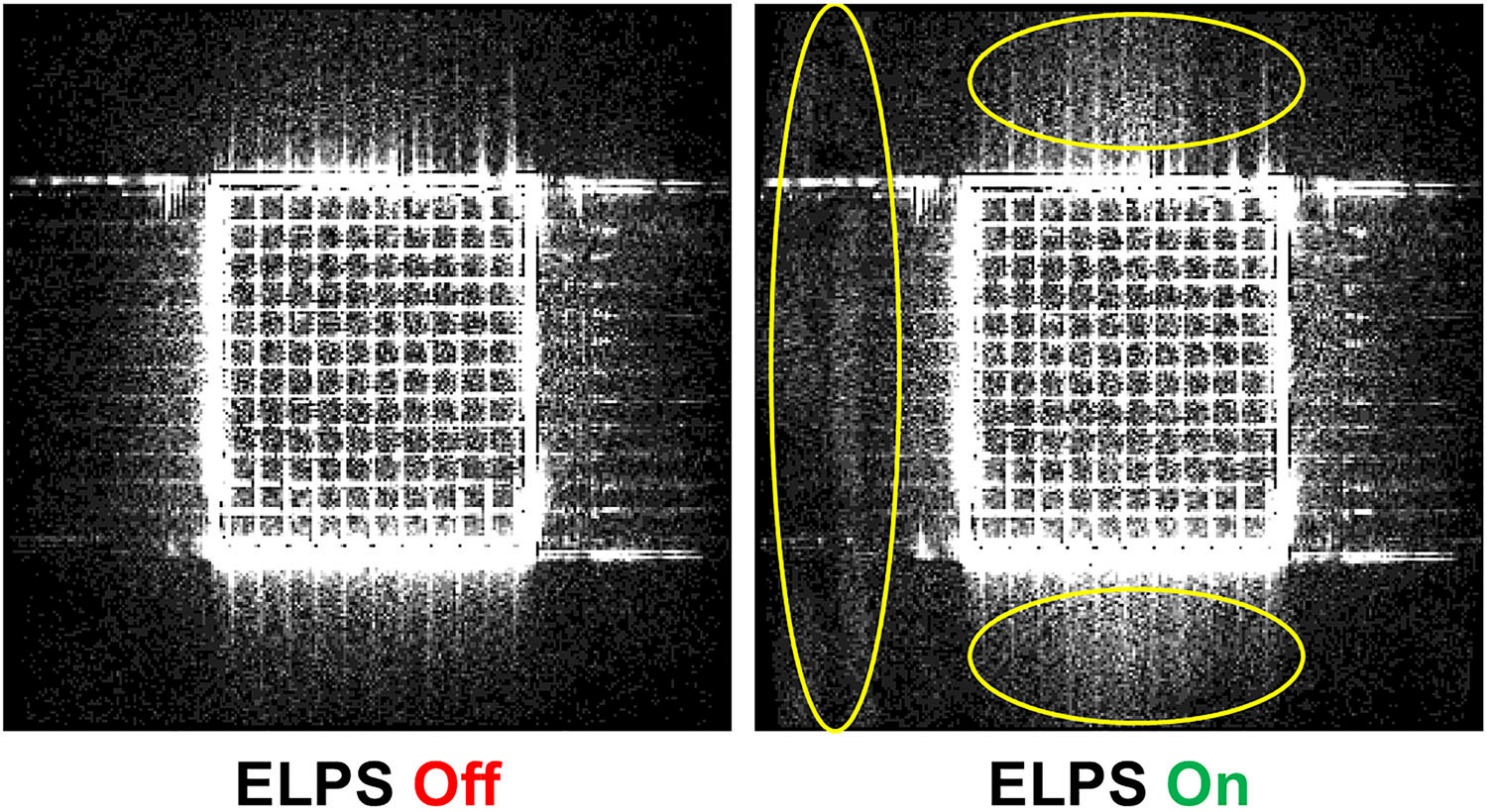
An RF interference artifact is seen with a low window/level setting on the left side of the image while increased general noise is noted in both the interior and superior regions.

A demonstration of the image quality in the QUASAR geometric distortion phantom is shown in Figure 2. Note how when the ELPS was activated, an RF interference noise band was seen on the left side of the image as well as on both the inferior and superior edges.

The quantitative geometric distortion analysis results from the vendor‐provided software is shown in Table 4. No substantial differences were noted between when the ELPS was deactivated compared to when it was activated.

**TABLE 4:** The results of the QUASAR analysis when the ELPS was both deactivated and activated.

	ELPS Deactivated	ELPS Activated
Absolute distortion mean (mm)	0.474	0.469
Absolute distortion standard deviation (mm)	0.143	0.142
Absolute distortion maximum (mm)	0.957	0.954
Absolute distortion percent above 0.8 mm (%)	3.147	3.247

### 3.2 Quantitative imaging analysis

**FIGURE 3:**
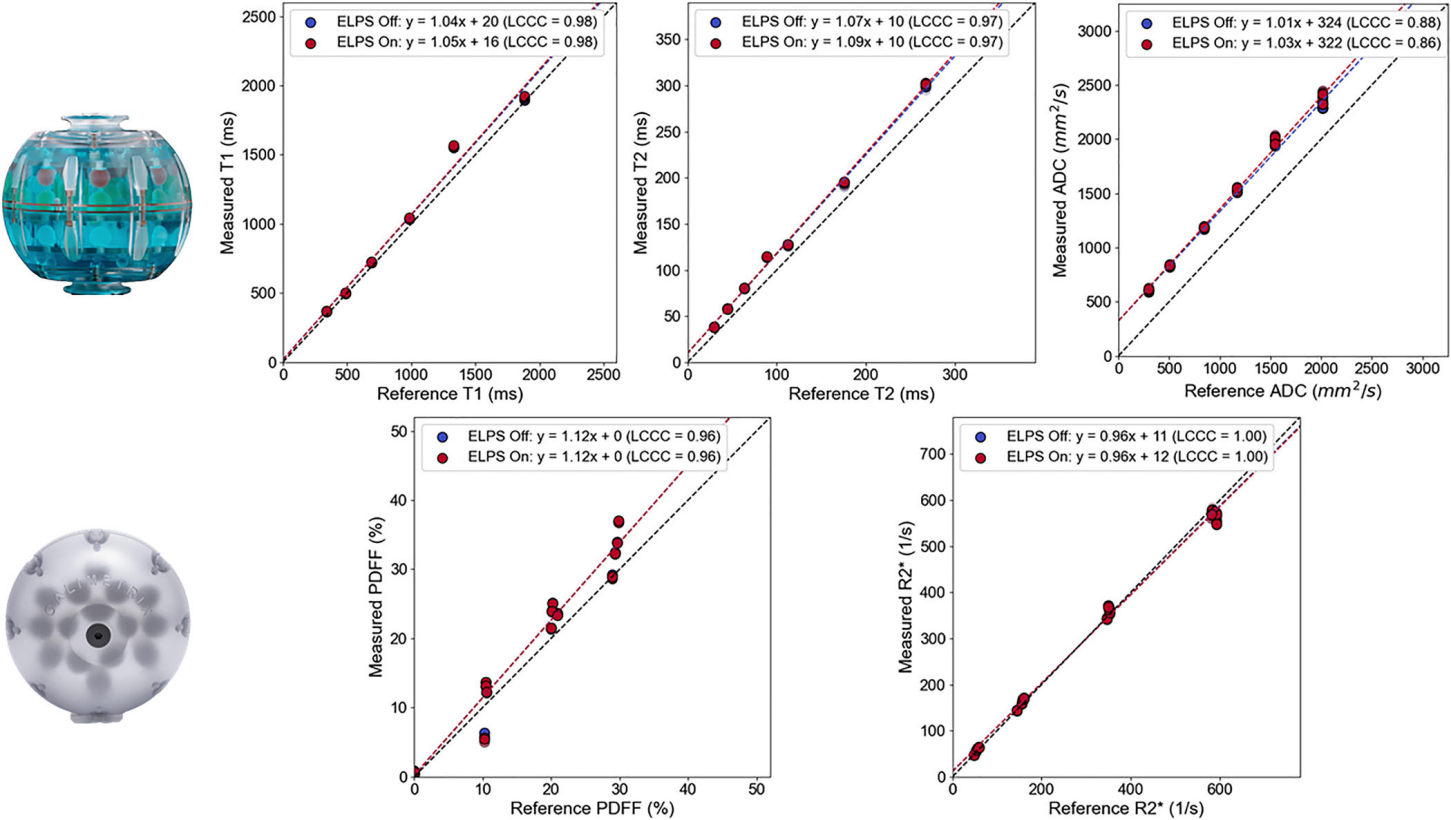
Plots of the agreement between the quantitative T1 (top left), T2 (top middle), ADC (top right), PDFF (bottom left), and R2* (bottom right) with corresponding linear lines‐of‐best‐fit and LCCC values for when the ELPS is both deactivated (blue) and activated (red).

Plots of the agreement between the measured mean values and reference values for the quantitative T1, T2, ADC, PDFF, and R2* for when the ELPS was both deactivated and activated are shown in Figure 3. Overall, there was minimal difference between quantitative measurements in terms of the linear line‐of‐best‐fit and the resulting LCCC when the ELPS is activated compared to when it was deactivated, however this could be due to the consolidation of the values by using the mean. Therefore, the next plots in this section will give a more detailed analysis of the results.

**FIGURE 4:**
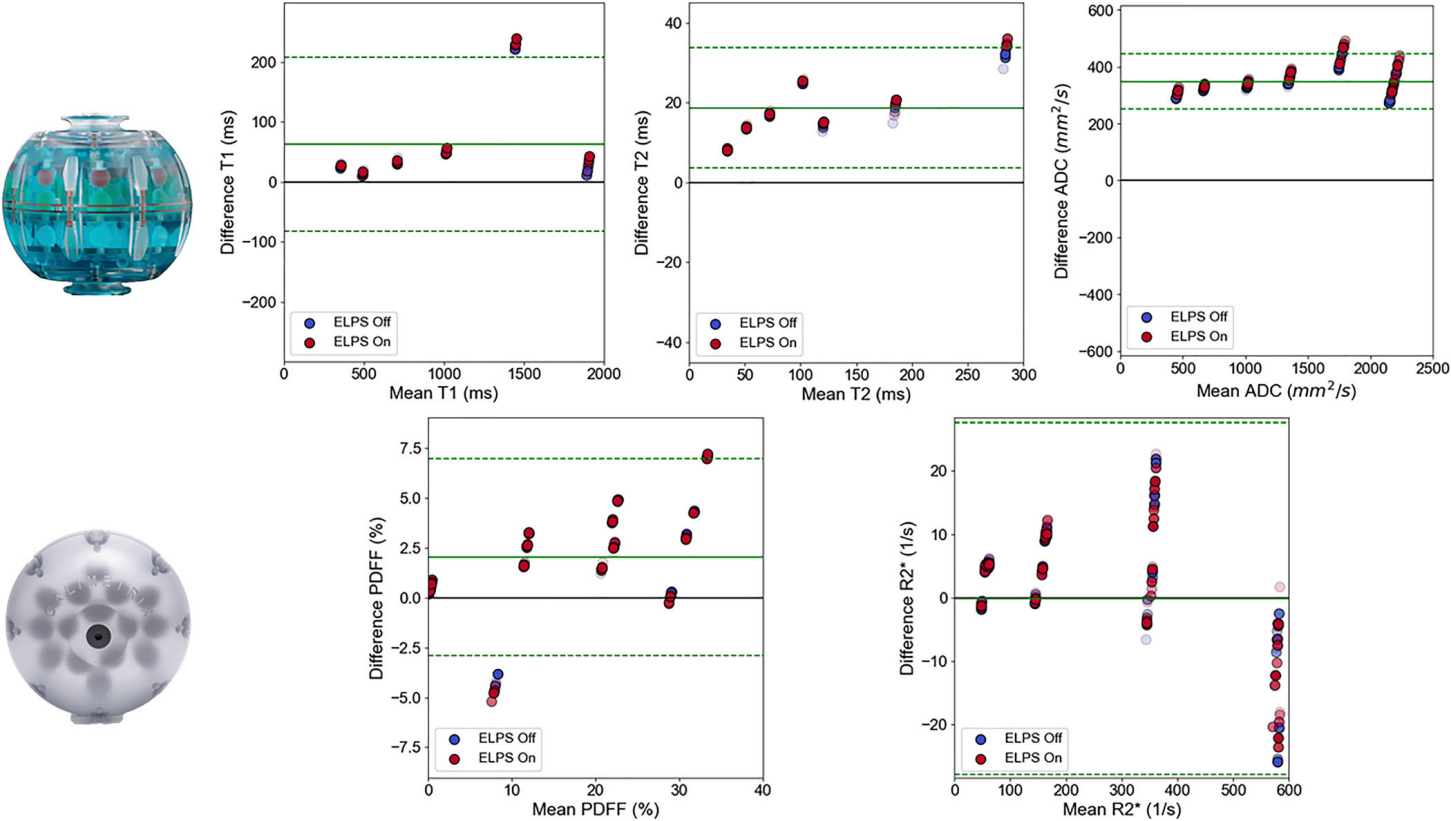
Bland‐Altman plots of the quantitative T1 (top left), T2 (top middle), ADC (top right), PDFF (bottom left), and R2* (bottom right) with corresponding mean (solid green) and 95% lines of agreement (dashed green) for when the ELPS is both deactivated (blue) and activated (red).

The respective Bland‐Altman plot for Figure 3 is shown in Figure 4. Similarly, little difference was seen between when the ELPS is activated and when it was deactivated. The difference between the highest measured quantitative T2 values against the reference values was slightly higher when the ELPS is activated, however the difference is minimal.

**FIGURE 5:**
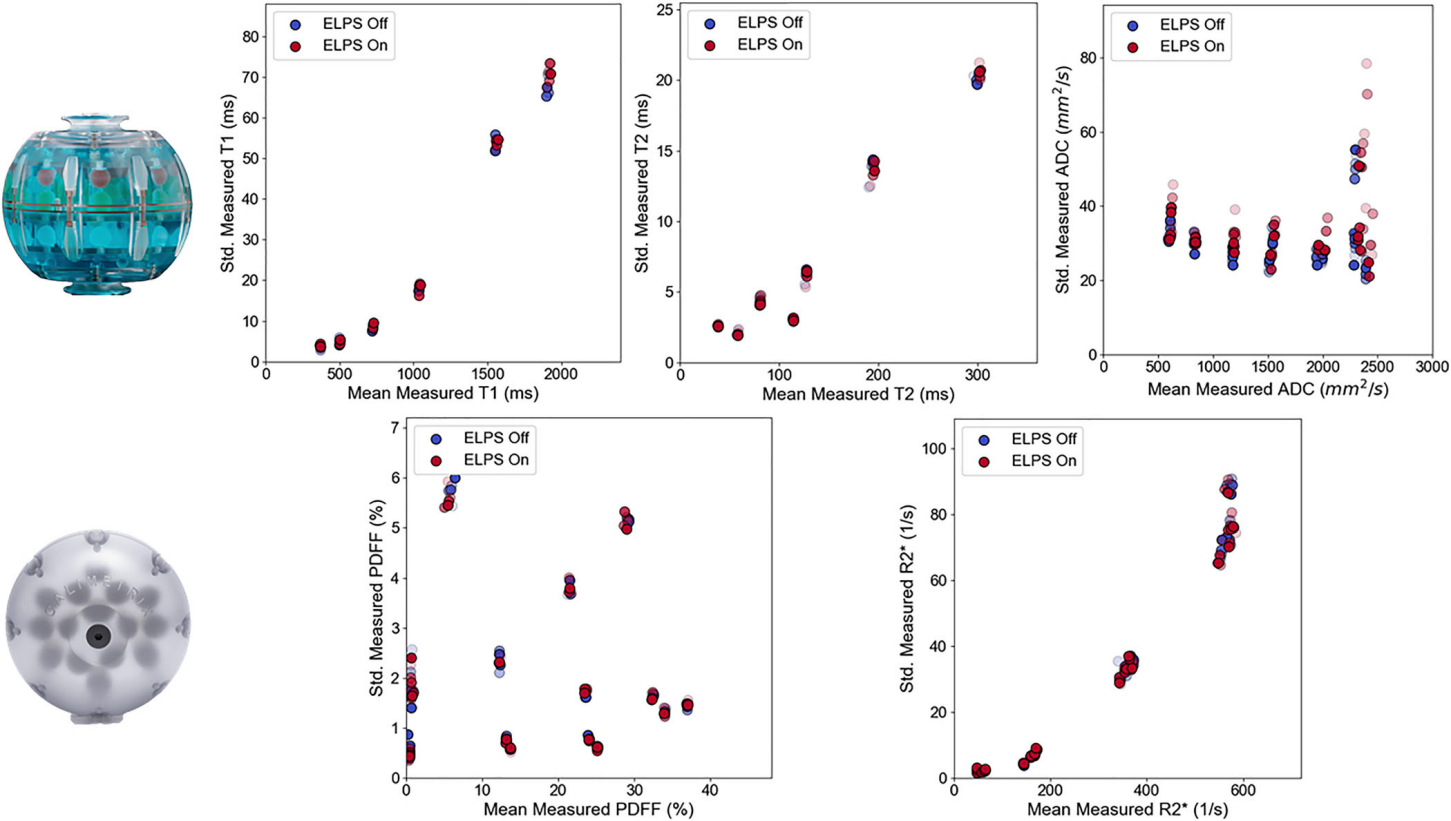
Plots of the standard deviation against the mean of the quantitative T1 (top left), T2 (top middle), ADC (top right), PDFF (bottom left), and R2* (bottom right) for when the ELPS is both deactivated (blue) and activated (red).

The largest difference between when the ELPS was activated compared to when it was deactivated is shown in Figure 5 which plots the standard deviation of the values inside each vial against their mean values. The largest difference was seen in the ADC which consistently demonstrates a higher standard deviation of the values inside the vial when the ELPS was activated.

**FIGURE 6:**
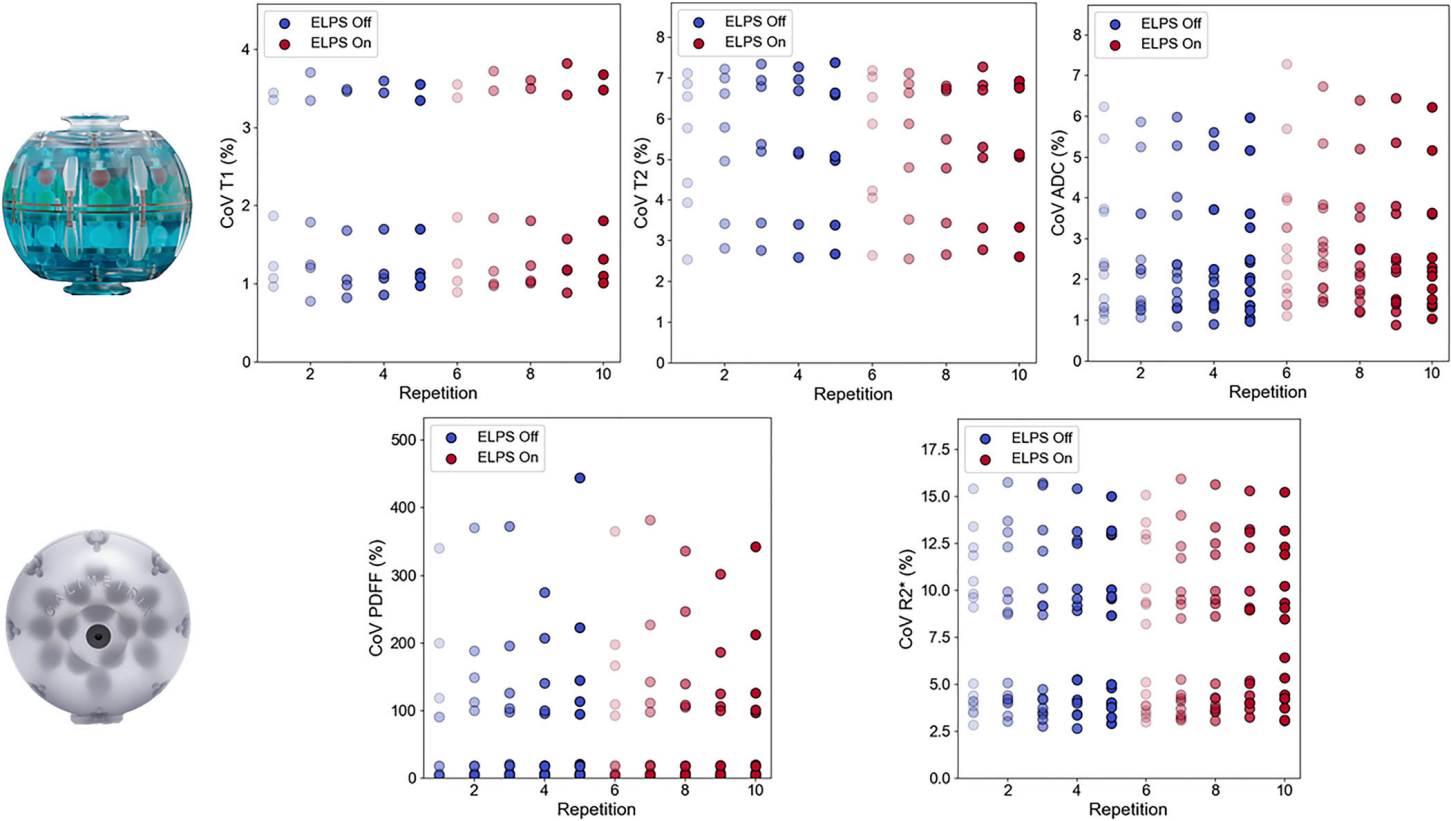
Plots of the CoV against the acquisition repetition for the quantitative T1 (top left), T2 (top middle), ADC (top right), PDFF (bottom left), and R2* (bottom right) for when the ELPS is both deactivated (blue) and activated (red).

To visualize Figure 5 more explicitly, Figure 6 demonstrates how the CoV changes across the quantitative values when the ELPS is activated compared to when it was previously deactivated during the repetitions. A notable increase was seen in the ADC values when the ELPS was activated compared to when it was deactivated, especially at the higher ADC values.

To investigate the increased standard deviation inside the phantom vials in the BLADE DWI sequence, the SNR difference between the individual b‐value images was determined to investigate the cause of this further. There were minimal SNR differences among the two b‐value images from when the ELPS is deactivated (77 and 26) and activated (75 and 26) for a b‐value of 0 s/mm^2^ and 800 s/mm^2^, respectively.

## 4 DISCUSSION

### 4.1 Summary

This study presented the effects of ELPS activation during MRI scanning on the resulting image quality across several coil combinations and quantification accuracy and repeatability of quantitative MRI sequences for T1, T2, ADC, PDFF, and R2* using clinically used coil combinations. This is the first known study to the author's knowledge to present such an analysis in detail. In summary, the novel contributions of this study to the characterization of ELPS activation during MRI scanning are: 1) the description of the SNR changes across different coil combinations, 2) the real‐time identification of the induced RF interference artifact using a cine acquisition, and 3) the resulting impacts on quantitative MRI accuracy and repeatability.

From the SNR analysis, the largest difference in SNR was in the integrated body coil due to the lowest SNR from the individual receive elements which gets increased with the higher channel coils such as the 18‐channel UltraFlex series and the dedicated anatomical coils (i.e., shoulder). This was most likely a contributing factor as to why there was little reported difference in quantitative values between when the ELPS deactivated and activated. However, degradation in image intensity uniformity, percent signal ghosting, and low contrast object detectability was seen during ACR Large Phantom testing using the 20‐channel Head/Neck coil.

The largest difference in the quantitative MRI sequences was seen in the ADC, which consistently demonstrated a higher standard deviation of the values inside the vial when the ELPS was activated. Since there was little difference in the SNR measurements among the individual b‐values images between when the ELPS was deactivated and activated, the difference in standard deviation may be due to noise propagation in specific vials affected the most. However, it should be noted that the difference in the CoV was also small at around 1% from 6% when the ELPS was deactivated to 7% when it was activated. It has been suggested that a mean ADC change of 7% is a significant predictor of differentiating head and neck cancer patients for both local control and recurrence‐free survival endpoints^21^. Therefore, the differences caused by the ELPS system as shown in this study would not be sufficient to affect this finding, however other applications should be carefully evaluated.

Possible explanations for the high level of agreement with both the MDME quantitative T1 and T2 maps are their acquisition parameter of a GRAPPA factor of 3 which has been shown to reduce noise in some areas while increasing noise in others^22^, ^23^. This effect could effectively nullify, or smear out, any association with electronic‐induced noise from the ELPS. However, the quantitative performance of the q‐Dixon sequence for PDFF and R2* was not affected even though it did not use any image acceleration techniques.

### 4.2 Limitations

The Calimetrix phantom used in this study has a maximum PDFF value of around 40% which limits its evaluation of fatty structures which are of interest in several anatomical sites such as in the head and neck where parotid salivary glands may exceed this value^24^. However, it should be noted that quantitative Dixon reconstruction algorithms should perform similarly at PDFF values symmetric around 50% due to the same fraction^25^ (i.e., 20% vs. 80%). In this study, we found a systematic positive ADC bias of ∼320 mm^2^/s which may be due to our use of BLADE DWI compared to a conventional echo planer imaging (EPI) technique which can lead to additional bias^5^. We determined that this bias was not due to incorrect temperature readings since the same temperature was used for the quantitative T1 and T2 values which did not experience a similar bias. We did, however, use the reference values provided by CaliberMRI instead of imaging a recommended ground‐truth quantitative sequence due to the possibility for bias compared to the reported reference values. On the other hand, our report primarily investigated the difference in quantitative values between when the ELPS was activated and deactivated instead of the accuracy of the quantitative measurements themselves. Additionally, our SNR calculation for the BLADE DWI individual b‐value images was done by using the CaliberMRI MiniHybrid phantom which does not contain a true homogeneous slice like the homogeneous phantom used in the study for the other acquisitions. On this note, the reported SNR values may not be truly representative of the actual image SNR since it is recommended to prevent including regions with artifacts into a noise measurement, whereas for this study they were purposefully included to quantify the effects of the ELPS system.

### 4.3 Future work

From a technical perspective, future studies should be done to characterize the ELPS‐induced RF interference artifact across different MRI system static magnetic field strengths and vendors. Our institution has a 1.5T Siemens MR‐simulation scanner, however we did not notice any noticeable level of ELPS‐induced RF interference artifact on that system since the ELPS system was nearly twice as far away from the MRI due to its increased room size. If true, this may be one of many considerations when site planning a new MRI system. Additionally, more robust image SNR analysis should be conducted to more accurately quantify the resulting SNR changes. It would be important to also evaluate the difference in a human subject with clinically used coil combinations, though from our results in phantoms, it should be unlikely to see a very large difference.

Since this study was conducted using only a single scanner and single ELPS system, independent quality assurance at commissioning should be done to accurately characterize the effects seen here. If similar concerning effects are seen, from an administrative and clinical workflow standpoint, the work shown here is motivational to persuade clinical staff with frequent opportunities to scan on an MRI‐simulation scanner (i.e., MRI technologists, physicists, etc.) to inform and remind their colleagues of the importance of deactivating the ELPS during scanning. Additionally, clinical supervisors should consider effective ways to properly educate clinical staff of the effects of the ELPS on the resulting MRI images, with their small but, importantly, preventable addition to the inherent noise in any image. It may be of interest to determine automatic ways to deactivate the ELPS during scanning such as how autoinjectors work during dynamic contrast‐enhanced (DCE) MRI examinations. Although the ELPS used in this study is MR Conditional dependent on being deactivated during MRI examination, there was a communication gap in our clinical workflow which prevented its deactivation during quality assurance testing. This recommendation from the vendor should therefore be properly carried forward to staff using its products.

## 5 CONCLUSION

This study demonstrated the effects of ELPS‐induced electronic noise interference on MRI image quality and quantification for T1, T2, ADC, PDFF, and R2* values. Although the integrated body coil demonstrated the most noticeable reduction in image quality, efforts should be made from the clinical team to ensure its deactivation during imaging to eliminate preventable reduction in SNR. Finally, although ELPS activation did not severely impact mean quantitative MRI sequence results, its CoV may be slightly affected, further reinforcing the need for the clinical team to address this issue. It should be reiterated that the entire ELPS must be deactivated instead of simply turning the visual laser to eliminate the production of the RF interference leading to the imaging artifacts.

## AUTHOR CONTRIBUTIONS

**Lucas McCullum**: Conceptualization; data curation; formal analysis; investigation; methodology; resources; software; validation; visualization; writing—original draft; writing—review and editing. **Yao Ding**: Data curation; methodology; resources; writing—review and editing. **Clifton D. Fuller**: Funding acquisition; resources; supervision; writing—review and editing. **Brian A. Taylor**: Conceptualization; data curation; funding acquisition; investigation; methodology; project administration; resources; supervision; validation; visualization; writing—review and editing.

## CONFLICT OF INTEREST STATEMENT

LM has received unrelated travel / hotel accommodations from Elekta AB. CDF has received unrelated grant support from Elekta AB and holds unrelated patents licensed to Kallisio, Inc. (US PTO 11730561) through the University of Texas, from which they receive patent royalties. CDF has also received unrelated travel and honoraria from Elekta AB, Philips Medical Systems, Siemens Healthineers/Varian, and Corewell Health. Additionally, CDF has served in an unpaid advisory capacity for Siemens Healthineers/Varian and has served on the guidelines/scientific committee for Osteoradionecrosis for the American Society of Clinical Oncology.

## ETHICS STATEMENT

The authors declare no ethics statement.
